# Application of validity theory and methodology to patient-reported outcome measures (PROMs): building an argument for validity

**DOI:** 10.1007/s11136-018-1815-6

**Published:** 2018-02-20

**Authors:** Melanie Hawkins, Gerald R. Elsworth, Richard H. Osborne

**Affiliations:** 1 Health Systems Improvement Unit, Centre for Population Health Research, Faculty of Health, Deakin University, Geelong, VIC 3220 Australia; 2 Department of Public Health, University of Copenhagen, Copenhagen, Denmark

**Keywords:** Patient-reported outcome measure, PROM, Validation, Validity, Interpretive argument, Interpretation/use argument, IUA, Validity argument, Qualitative methods, Health literacy, Health Literacy Questionnaire (HLQ)

## Abstract

**Background:**

Data from subjective patient-reported outcome measures (PROMs) are now being used in the health sector to make or support decisions about individuals, groups and populations. Contemporary validity theorists define validity not as a statistical property of the test but as the extent to which empirical evidence supports the interpretation of test scores for an intended use. However, validity testing theory and methodology are rarely evident in the PROM validation literature. Application of this theory and methodology would provide structure for comprehensive validation planning to support improved PROM development and sound arguments for the validity of PROM score interpretation and use in each new context.

**Objective:**

This paper proposes the application of contemporary validity theory and methodology to PROM validity testing.

**Illustrative example:**

The validity testing principles will be applied to a hypothetical case study with a focus on the interpretation and use of scores from a translated PROM that measures health literacy (the Health Literacy Questionnaire or HLQ).

**Discussion:**

Although robust psychometric properties of a PROM are a pre-condition to its use, a PROM’s validity lies in the sound argument that a network of empirical evidence supports the intended interpretation and use of PROM scores for decision making in a particular context. The health sector is yet to apply contemporary theory and methodology to PROM development and validation. The theoretical and methodological processes in this paper are offered as an advancement of the theory and practice of PROM validity testing in the health sector.

## Background

The use of patient-reported outcome measures (PROMs) to inform decision making about patient and population healthcare has increased exponentially in the past 30 years [1–8]. However, a sound theoretical basis for validation of PROMs is not evident in the literature [2, 9, 10]. Such a theoretical basis could provide methodological structure to the activities of PROM development and validity testing [10] and thus improve the quality of PROMs and the decisions they help to make.

The focus of published validity evidence for PROMs has been on a limited range of quantitative psychometric tests applied to a new PROM or to a PROM used in a new context. This quantitative testing often consists of estimation of scale reliability, application of unrestricted factor analysis and, increasingly, fitting of a confirmatory factor analysis (CFA) model to data from a convenience sample of typical respondents. The application of qualitative techniques to generate target constructs or to cognitively test items has also become increasingly common. However, contemporary validity testing theory emphasises that validity is not just about item content and psychometric properties; it is about the ongoing accumulation and evaluation of sources of validity evidence to provide supportive arguments for *the intended interpretations and uses of test scores in each new context* [10–12], and there is little evidence of this thinking being applied in the health sector [10].

While there are authors who have provided detailed descriptions of PROM validity testing procedures [13–15], there are few publications that describe the iterative and comprehensive testing of the validity of the interpretations of PROM data for the intended purposes [10]. This gap in the research is important because validity extends beyond the statistical properties of the PROM [10, 16, 17] to the veracity of interpretations and uses of the data to make decisions about individuals and populations [10, 11]. In keeping with the advancement of validity theory and methodology in education and psychology [11], and with application to the relatively new area of measurement of patient-reported outcomes in health care, a more comprehensive and structured approach to validity testing of PROMs is required.

There is a strong and long history of validation theory and methodology in the fields of education and psychology [12, 18–22]. Education and psychology use many tests that are measures of student or patient objective and subjective outcomes and progress, and these disciplines have been required to develop sound theory and methodology for validity testing of not only the measurement tools but of how the data are interpreted and used for making decisions in specified contexts [11, 23]. The primary authoritative reference for validity testing theory in education and psychology is the *Standards for Psychological and Educational Testing* [11] (hereon referred to as the *Standards*)^1^. It advocates for the iterative collection and evaluation of sources of validity evidence for the interpretation and use of test data in each new context [11, 24]. The validity testing theory of the *Standards* can be put into practice through a methodological framework known as the argument-based approach to validation [12, 23]. Validation theorists have debated and refined the argument-based approach since the middle of the twentieth century [18–20, 25, 26].

The valid interpretation of data from a PROM is of vital importance when the decisions will affect the health of an individual, group or population [27]. Psychometrically robust^2^ properties of a measurement tool are a pre-condition to its use and an important component of the validity of the inferences drawn from its data in its development context but do not guarantee valid interpretation and use of its data in other contexts [10, 28, 29]. This is particularly the case, for example, for a PROM that is translated to another language because of the risk of poor conversion of the intent of each item (and thus the construct the PROM aims to measure) into the target language and culture [30]. The aim of this paper is to apply contemporary validity testing theory and methodology to PROM development and validity testing in the health sector. We will give a brief history of validity testing theory and methodology and apply these principles to a hypothetical case study of the interpretation and use of scores from a translated PROM that measures the concept of health literacy (the Health Literacy Questionnaire or HLQ).

## Validity testing theory and methodology

### Validity testing theory

Iterations of the *Standards* have been instrumental in establishing a clear theoretical foundation for the development, use and validation of tests, as well as for the practice of validity testing. The *Standards* (2014) defines validity as ‘the degree to which evidence and theory support the interpretations of test scores for proposed uses of tests’ (p. 11), and states that ‘the process of validation involves accumulating relevant evidence to provide a sound scientific basis for the proposed score interpretations’ (p. 11) [11]. It also emphasises that the proposed interpretation and use of test scores must be based on the constructs the test purports to measure (p. 11). This paper is underpinned by these definitions of validity and the process of validation, and the view that construct validity is the main foundation of test development and interpretation for a given use [31].

Early thinkers about validity and testing defined validity of a test through correlation of test scores with an external criterion that is related to the purpose of the testing, such as gaining a particular school grade for the purpose of graduation [32]. During the early part of the twentieth century, statistical validation dominated and the focus of validity came to rest on the statistical properties of the test and its relationship with the criterion. However, there were problems with identifying, defining and validating the criterion with which the test was to be correlated [33], and it was from this dilemma that the notions of content and construct validity arose [22].

Content validity is how well the test content samples the subject of testing, and construct validity refers to the extent to which the test measures the constructs that it claims to measure [18, 25]. This thinking marked the beginning of the movement that advocated that multiple lines of validity evidence were required and that the purpose of testing needed to be accounted for in the validation process [18, 34]. In 1954 and 1955, the first technical recommendations for psychological and educational achievement tests (later to become the *Standards*) were jointly published by the American Educational Research Association (AERA), American Psychological Association (APA) and the National Council on Measurement in Education (NCME), and these promoted predictive, concurrent, content and construct validities [32, 34–36]. As validity testing theory evolved, so did the APA, AERA and NCME *Standards* to progressively include (a) notions of user responsibility for validity of a test embodied in three types of validity—criterion (predictive + concurrent), content and construct [37, 38]; (b) that construct validity subsumes all other types of validity to form the unified validity model [25, 38–42]; and (c) that it is not the test that is validated but the score inferences for a particular purpose, with additional concern for the potential social consequences of those inferences [11, 19, 43, 44]. Consideration of social consequences brought the issue of fairness in testing to the forefront, the concept of which was first included as a chapter in the 1999 *Standards*: Chap. 7. Fairness in testing and test use (p. 73). The 1999 and 2014 versions of the *Standards* also recognised the notion of argument-based validation [12, 19, 23]. Validation of a test for a particular purpose is about establishing an argument (that is, evaluating validity evidence) not only for the test’s statistical properties, but also for the inferences made from the test’s scores, and the actions taken in response to those inferences (the consequences of testing) [23, 25, 33, 41, 45–47].

In conceptualising validation practice, the *Standards* outlines five sources of validity evidence [11]:

1. Evidence based on the *content* of the test (i.e. relationship of item themes, wording and format with the intended construct, and administration including scoring)
2. Evidence based on the *response processes* of the test (i.e. cognitive processes, and interpretation of test items by respondents and users, as measured against the intended interpretation or construct definition)
3. Evidence based on the test’s *internal structure* (i.e. the extent to which item interrelationships conform to the constructs on which claims about the score interpretations are based)
4. Evidence based on the *relationship of the scores to other variables* (i.e. the pattern of relationships of test scores to external variables as predicted by the construct operationalised for a specific context and proposed use)
5. Evidence for *validity and the consequences of testing* (i.e. the intended and unintended consequences of test use, and as traced to a source of invalidity such as construct underrepresentation or construct-irrelevant components).

These five sources of evidence demand comprehensive and cohesive quantitative and qualitative validity evidence from development of the test through to establishing the psychometric properties of the test and to the interpretation, use and consequences of the score interpretations [11, 48, 49]. As is also outlined in the *Standards* (2014, p. 23–25), it is critical that a range of validity evidence justify (or argue for) the interpretation and use of test scores when applied in a context and for a purpose other than that for which the test was developed.

### Validity testing methodology

The theoretical framework of the 1999 and 2014 *Standards* was strongly influenced by the work of Kane [12, 23, 45, 50, 51]. Kane’s argument-based approach to validation provides a framework for the application of validity testing theory [12, 23, 52]. The premise of this methodology is that ‘validation involves an evaluation of the credibility, or plausibility, of the proposed interpretations and uses of test scores’ (p. 180) [51]. There are two steps to the approach:

1. Develop an *interpretive argument* (also called the interpretation/use argument or IUA) for the proposed interpretations of test scores for the intended use, and the assumptions that underlie it: that is, clearly, coherently and completely outline the proposed interpretation and use including, for example, context, population of interest, types of decisions to be made and potential consequences, and specify any associated assumptions;
2. Construct a *validity argument* that evaluates the plausibility of the interpretive argument (i.e. the interpretation/use claims) through collection and analyses of validation evidence: that is, assess the evidence to build an argument for, or perhaps against, the proposed interpretation and use of test scores.

**Fig. 1:**
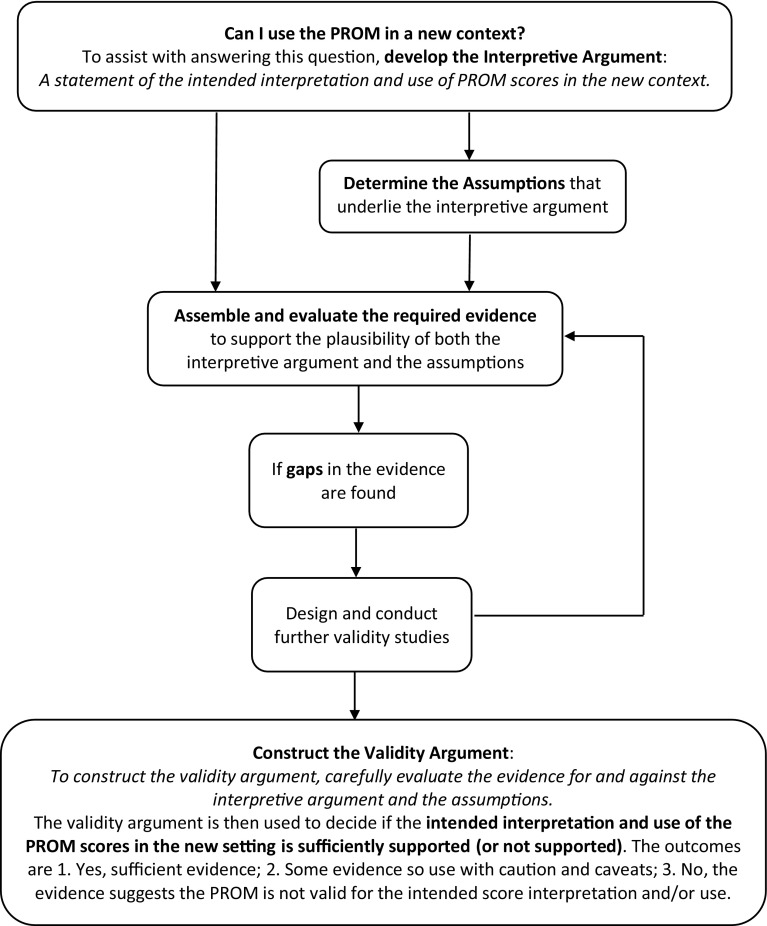
Flow chart of the application of validity testing theory and methodology to assess the validity of patient-reported outcome measure (PROM) score interpretation and use in a new context

As shown in Fig. 1, a validity argument is developed through evaluation of the available evidence and, if necessary, the generation of new evidence. Available evidence for the validity of the use of PROM data to make decisions about healthcare is usually in the form of publications about the development and applications of the PROM. However, further research will frequently be required to test the PROM for a new purpose or in a new context. Evaluation of evidence for assumptions that might underlie the interpretive argument may also be required. For example, consider that a PROM will be translated from a local language to the language of an immigrant group and will be used to compare the health literacy of the two groups. A critical assumption underpinning this comparison is that there is measurement equivalence between the two versions of the PROM. This assumption will require new evidence to support it. As we have outlined, the *Standards* specifies five sources of validity evidence that are required, as appropriate to the test and the test’s purpose.

While some quantitative psychometric information is usually available for most tests [10, 53], collection of evidence that the PROM captures the constructs it was designed to capture, and that these constructs are appropriate in new contexts, will require qualitative methods, as well as quantitative methods. Qualitative methods can, for instance, ascertain differences in response (i.e. cognitive) processes or interpretations of items or scores across respondent groups or users of the data, and whether or not new language versions of a measurement tool capture the item intents (and thus the construct criteria) of the source language tool [6, 16, 17, 41, 50]. For many tests, there is little published qualitative validity evidence even though these methods are critical to gaining an understanding of the validity of the inferences made from PROM data [10, 17, 41]. Additionally, there are almost no citations of the most authoritative reference for validity theory, the *Standards*: ‘…despite the wide-ranging acknowledgement of the importance of validity, references to the *Standards* is [sic] practically non-existent. Furthermore, many validation studies are still firmly grounded in early twentieth century conceptions that view validity as a property of the test, without acknowledging the importance of building a validity argument to support the inferences of test scores’ (p. 340) [10].

### The Health Literacy Questionnaire (HLQ)

By way of example, we now use a widely used multidimensional health literacy questionnaire, the HLQ, to illustrate the development of an interpretive argument and corresponding evidence for a validity argument. The HLQ was informed by the World Health Organization definition of health literacy: *the cognitive and social skills which determine the motivation and ability of individuals to gain access to, understand and use information in ways which promote and maintain good health* [54]. While validity testing of the HLQ has been conducted in English-speaking settings [55–59], evidence for the use of translated versions of the HLQ in non-English-speaking settings is still being collected [60–62].

In short, the HLQ consists of 44 items within nine scales, each scale representing a unique component of the multidimensional construct of health literacy. It was developed using a grounded, validity-driven approach [31, 63] and was initially developed and tested in diverse samples of individuals in Australian communities. Initial validation of the use of the HLQ in Australia has found it to have strong construct validity, reliability and acceptability to clients and clinicians [55]. Items are scored from 1 to 4 in the first 5 scales (Strongly Disagree, Disagree, Agree, Strongly Agree), and from 1 to 5 in scales 6–9 (Cannot Do or Always Difficult, Usually Difficult, Sometimes Difficult, Usually Easy, Always Easy). The HLQ has been in use since 2013 [56, 57, 64–72] and was designed to furnish evidence that would help to guide the development and evaluation of targeted responses to health literacy needs [64, 73]. Typical decisions made from interpretations of HLQ data are those to do with changes in clinical practice (e.g. to enable clinicians to better accommodate patients with high health literacy needs); changes an organisation might need to make for system improvement (e.g. access and equity); development of group or population health literacy interventions (e.g. to develop policy for population-wide health literacy intervention); and whether or not an intervention improved the health literacy of individuals or groups.

Translated HLQ scales are expected by the HLQ developers and by the users of a translated HLQ to measure the same constructs of health literacy in the same way as the English HLQ. The English HLQ is translated using the Translation Integrity Procedure (TIP), which was developed by two of the present authors (MH, RHO) in support of the wide application of the HLQ and other PROMs [74, 75]. The TIP is a systematic data documentation process that includes high/low descriptors of the HLQ constructs, and descriptions of the intended meaning of each item (item intents). The item intents provide translators with in-depth information about the intent and conceptual basis of the items and explanations of or synonyms for words and phrases within each item. The descriptions enable translators to consider linguistic and cultural nuances to lay the foundation for achieving acceptable measurement equivalence. The item intents are the main support and guidance for translators, and are the primary focus of the translation consensus team discussions.

### An example of an interpretive argument for a translated PROM

**Fig. 2:**
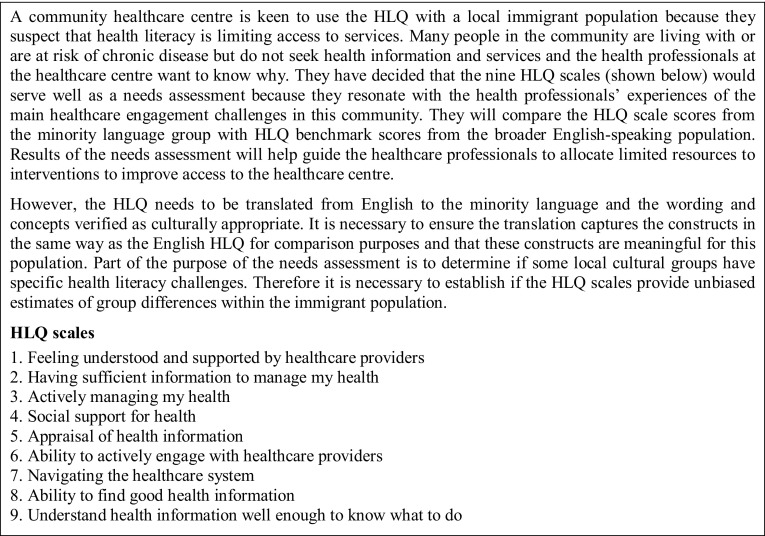
Community Healthcare Centre Vignette: a community healthcare centre wishes to use the HLQ as a community needs assessment for a minority language group

An interpretive argument is a statement of the proposed interpretation of scores for a defined use in a particular context. The role of an interpretive argument is to make clear how users of a PROM intend to interpret the data and the decisions they intend to make with these data. Underlying the interpretive argument are often embedded assumptions. Evidence may exist or may need to be generated to justify these assumptions. In this section, we describe an interpretive argument, and associated assumptions, for the potential interpretation and use of data from a translated HLQ in a hypothetical case of a community healthcare centre that seeks to understand and respond to the health literacy strengths and challenges of its client population (see Fig. 2. A Community Healthcare Centre Vignette).

#### The interpretive argument (interpretation and use of scores)

For this example, the HLQ scale scores will provide data about the health literacy needs of the target population of the community healthcare centre and will be *interpreted* according to the HLQ item intents and high/low descriptors of the HLQ constructs, as described by the HLQ authors [55]. Appropriately normed scale scores will indicate areas in which different immigrant sub-groups are less or more challenged in terms of health literacy and will be *used* by the healthcare managers to make decisions about resource allocation to interventions to improve access to the healthcare centre.

#### Assumptions underlying the interpretive argument

The interpretive argument *assumes* there is an appropriate range of sound empirical evidence for the development and initial validity testing of the English HLQ and for the HLQ translation method.

The assumption that there is sound validity evidence for the source language PROM and for the PROM translation process is the foundation for an interpretive argument for any translated PROM. Although it could be possible for a good translation process to improve items during translation (by, for example, removing ambiguous or double barrelled items), it is important that a translation begins with a PROM that has undergone a sound construction process, has acceptable statistical properties, and for which there is a strong validity argument for the interpretation and use of its data in the source language. Conversely, a poor or even unintentionally remiss translation can take a sound PROM and produce a translated PROM that does not measure the same constructs in the same way as the source PROM, and which may lead to misleading or erroneous data (and thus misleading or erroneous interpretations of the data) about individuals or populations to which the PROM is applied.

### Constructing a validity argument for a translated PROM

A validity argument is an evaluation of the empirical evidence for and against the interpretive argument and its associated assumptions. This is an iterative process that draws on the relevant results of past studies and, if necessary, guides further studies to yield evidence to establish an argument for new contexts. If the interpretive argument and assumptions are evaluated as being comprehensive, coherent and plausible, then it may be stated that the intended interpretation and use of the test scores are valid [45] until or unless proven otherwise. Depending on the intended interpretation and use of the scores of a PROM—for example, as a needs assessment, a pre-post measure of a health outcome in a target population group, for health intervention development, or for cross-country comparisons—certain types of evidence will prove more necessary, relevant or meaningful than others to support the interpretive argument [28].

The five categories of validity evidence in the *Standards*, as well as the argument-based approach to validation, provide theoretical and methodological platforms on which to systemically formulate a validation plan for a new PROM or for the use of a PROM in a new context. When a PROM is translated to another language, the onus is on the developer or user of the translated PROM to methodically accumulate and evaluate validity evidence to form a plausible validity argument for the proposed interpretation and use of the PROM scores [11]. In our example of a translated HLQ used for health literacy needs assessment and to guide intervention development, a validity argument could include evaluation of evidence that:

- Supports sound initial HLQ construction and validity testing.
- The HLQ items and response options are appropriate for and understood as intended in the target culture.
- There is replication of the factor structure and measurement equivalence across sociodemographic groups and agencies in the target culture.
- The HLQ scales relate to external variables in anticipated ways in the target culture, both to known predictor groups (e.g. age, gender, number of comorbidities) and to anticipated outcomes (e.g. change after effective interventions).
- There is conceptual and measurement equivalence of the translated HLQ with the English HLQ, which is necessary to transfer the meaning of the constructs for interpretation in the target language [76] such that intended benefits of testing are more likely attained.

Our example case draws attention to (1) the need for the rigorous construction and initial validation methods of a source language PROM to ensure acceptable statistical properties, and (2) the need for a high-quality translation, evidence for which contributes to the validity argument for a translated PROM. Without these two factors in place, interpretations of data from a translated PROM for any purpose may be rendered unreliable.

Evidence, organised according to the five sources of validity evidence outlined in the *Standards*, for both the interpretive argument and the assumptions constitutes the validity argument for the use of the translated HLQ in this new language and context. In Table 1, column 1 displays components of the interpretive argument and assumptions to be tested; column 2 displays the evidence required for the components in column 1 and expected as part of a validity argument for a translated PROM in a new language/cultural context; and column 3 displays examples of methods to obtain validity data, including reference to relevant HLQ studies. When methods are described in general terms (e.g. cognitive interviews, confirmatory factor analysis), one method may be suggested for generating data for more than one source of evidence. However, the research participants or the focus of specific analyses will vary according to the nature of the evidence required. Table 1 serves as both a general guide to the theoretical logic of the *Standards* for assembling evidence to support the validity of inferences drawn from a newly translated PROM and as an outline of the published evidence that is available for some HLQ translations. However, establishing a validity argument for a PROM involves not just the accumulation of publications (or other evidence sources) about a PROM; it requires the PROM user to evaluate those publications and other evidence to determine the extent and quality of the existing validity testing (and how it relates to use in the intended context), and to determine areas if and where further testing is required [10]. Given that our case of a translated PROM is hypothetical, we do not provide a validity argument from (hypothetical) evaluated evidence. While a wide range of evidence has been generated for the original English HLQ, only some evidence has been generated for the use of translated HLQs in some specific settings [60–62, 77, 78]; therefore, the accumulation of much more evidence is warranted. The publications and examples that are cited in Table 1 provide guidance for the types of studies that could be conducted by users of translated PROMs and also indicate where evidence for translated HLQs is still required.

**Table 1:** Evaluating validity evidence for an interpretive argument for a translated patient-reported outcome measure (PROM)

Components of the interpretive argument and assumptions	Evidence required for a validity argument	Examples of methods to obtain validity data, including relevant studies on the Health Literacy Questionnaire (HLQ)
**1. Test content** [11]*—*analysis of test development (relationship of item themes, wording and format with the intended construct) and administration (including scoring)		
1.1 The content of the source language constructs and items are appropriate for the target culture	Evidence that the PROM constructs are appropriate and relevant for members of the target culture and that item content will be understood as intended by members of the target culture	Pre-translation qualitative evaluation by in-context PROM user about the cultural appropriateness of the items and constructs for the target language and culture. For example, an ethnographer who lived with Roma populations assessed each of the HLQ scales according to how Roma people might understand them [79]. The outcome was a decision that the HLQ scales, specifically as used in needs assessment for the Ophelia process [73], resonate with elements of successful grassroots health mediation programs in Roma communities
1.2 Application of a systematic translation protocol that provides evidence that linguistic equivalence and cultural appropriateness are highly likely to be achieved, thus supporting the argument for the successful transfer of the intended meaning of the source language constructs while maximising understanding of items, response options and administration methods in the target language and culture Construct irrelevance and construct underrepresentation in the target culture are considered	Evidence that a structured translation method, with detailed descriptions of the item intents, was appropriately implemented Evidence for the effective engagement of people from the target language/culture in the translation process Evidence of the confirmation of congruence of items and constructs between the source language and culture and the target language and culture to ensure construct relevance and avoid possible construct underrepresentation	Formal and documented translation method and process to manage translations to different languages, including documentation of participants in translation consensus meetings. A developer or other person deeply familiar with the PROM’s content and purpose oversees the concordance between the intents of original items and target language items. For example, two papers that describe the translation of the HLQ to other languages (Slovak [62] and Danish [60]) discuss the method of translation including the use of HLQ item intents, and they list the participants who took part in the detailed analysis of the translated items to collectively decide on the final items Systematic recording of steps in the translation and in the analysis of the recorded data, including changes made through the process In-depth cognitive interviews with target audience in the target culture about the items on the translated PROM, and content analysis to compare narrative data from interviews with the source language PROM item intents and construct definitions
**2. Response processes** [11]*—*cognitive processes, and interpretation of test items by respondents and users, as measured against the intended interpretation or construct definition		
2.1 Respondents to both the translated PROM and source language PROM engage in the same or similar cognitive (response) processes when responding to items, and these processes align with the source language construct criteria, thus indicating that similar respondents across cultures are formulating responses to the same items in the same way	Evidence that linguistic equivalence and cultural appropriateness has been achieved in the PROM translation Evidence that the cognitive processes of respondents match construct criteria (i.e. respondents to the translated PROM are engaging with the criteria of the source language construct when answering items) and that the intended understanding of items has been achieved	Analysis of the documented translation process to determine how difficulties in translation were resolved such that each translated item retained the intent of its corresponding source language item, while accommodating linguistic and cultural nuances. For example, in the German HLQ publication, the translation method is described, as well as linguistic and cultural adaptation difficulties encountered and how these were resolved [61] Cognitive interviews with target audience in the target culture to determine how respondents formulate their answers to items (i.e. assessment of the alignment of cognitive processes with source construct criteria). For example, Maindal et al. [60] conducted interviews to test the cognitive processes of target respondents when answering the Danish HLQ items, which was a way to test if the translated items were understood as intended. Another example of cognitive testing, although not a study of translated HLQ items, is that done by Hawkins et al. [56] to determine concordances/discordances between patient and clinician HLQ responses about the patient, and to match these with the HLQ item intents. This could be a useful method to determine if the intent of translated items and scales is understood in the same way by both patients and clinicians in a new cultural setting. This is important because clinician understanding about differences in perspectives about the construct being measured (in this case health literacy) may facilitate discussions that help clinicians better understand a patient’s perspective about their health, and therefore make better decisions about the patient’s care. These qualitative interview data can then be integrated with statistical analysis of the measurement equivalence (or Differential Item Functioning (DIF) analysis) of data between target and source language PROMs (Chap. 11, pp. 193–209) [5]
2.2 PROM users (e.g. health professionals or researchers who administer the PROM and interpret the PROM scores) of both the translated and source language PROMs engage in the same or similar cognitive processes (i.e. apply source language construct-relevant criteria) when interpreting scores, and that these processes match the intended interpretation of scores	Evidence that the cognitive processes of PROM users when evaluating respondents’ scores from a translated PROM are consistent with the source language construct criteria and with the interpretation of scores as intended by developers of the source language PROM	In-depth cognitive interviews with target PROM users, and content analysis to compare narrative data from interviews with source language PROM item intents and construct definitions, and to compare narrative data with the data from cognitive interviews with source language PROM users. For example, the Hawkins et al. study [56] shows how important it is that clinicians apply the same/similar construct criteria when interpreting HLQ scores as patients have applied when answering the items. Cognitive interviews to test response processes of the target audience of the translated PROM should also be conducted with the users of the PROM data who will interpret and use the scores to make decisions about those target audience members
**3. Internal structure** [11]*—*the extent to which item interrelationships conform to the constructs on which claims about the score interpretations are based		
3.1 Item interrelationships and measurement structure of scales of the translated PROM conform to the constructs of the source language PROM The constructs of translated PROMs in diverse cultures are thus conceptually comparable, and interpretations based on statistical comparisons of scale scores are unbiased	Evidence that the translated PROM scales are homogeneous and distinct and thus items are uniquely related to the hypothesised target constructs Evidence to confirm that the measurement structure of the source language PROM has been maintained through the translation process Evidence of measurement equivalence between the constructs of translated and source language PROMs	Confirmatory factor analysis (CFA) of data in the target language culture and comparisons with CFAs from data in the source language culture Reliability of the hypothesised PROM scales in the target culture For example, studies of the HLQ translated into Danish [60], German [61] and Slovak [62] present and discuss psychometric results that confirm the nine-factor structure of the HLQs and present fit statistics, factor loading patterns, inter-factor correlations and reliability estimates of the individual scales that are comparable to those found in the original development and replication studies [55, 58] DIF or, equivalently, multi-group factor analysis studies (MGFA) to establish configural, metric and scalar measurement equivalence of source and translated scales
**4. Relations to other variables** [11]*—*the pattern of relationships of test scores to external variables as predicted by the construct operationalised for a specific context and proposed use		
4.1 Convergent-discriminant validity is established for the translated PROM	Evidence that the relationships between the translated PROM and similar constructs in other tools are substantial and congruent with patterns observed in the source PROM (i.e. convergent evidence) such that score interpretation of the translated PROM is consistent with the score interpretation of the source language PROM and other PROMs measuring similar constructs Evidence that relationships between items and scales in the translated PROM and items and scales measuring unrelated constructs of tools in the broader domain of interest (e.g. health-related beliefs and attitudes more generally) are low (i.e. discriminant evidence)	Use of CFA to examine Fornell and Larker’s [80, 81] criteria for convergent-discriminant validity to compare translated PROM scales with measures of similar and contrasting constructs with other tools within the domain of interest; also comparison of these scale relationships across source and target cultures. Elsworth et al. used these criteria to assess convergent/discriminant validity of the HLQ *within* this multi-scale PROM [58]. This method could similarly be applied to a study of the relationships of a single or multi-scale health literacy PROM *across* similar health literacy assessments and PROMs assessing divergent health-related constructs Possible multitrait-multimethod (MTMM) studies of the translated PROM scales and other measures in the relevant domain [82]
4.2 Test–criterion relationships are robust for translated PROMs	Evidence that test–criterion relationships are concordant with expectations from theory to provide general support for construct meaning and information to support decisions about score interpretation and use for specific population groups and purposes Evidence that supports theoretically indicated equalities and differences in the distribution of scale scores across cultures to further support scale interpretation and use in target cultures	Correlation and group differences, e.g. analysis of variance of translated PROM summed scores by sub-groups (i.e. gender, age, education etc.) Multi-group CFA (MGCFA) by sub-groups. For example, Maindal et al. [60] investigated relationships between the scales of the newly translated Danish HLQ and a range of sociodemographic variables (e.g. gender, age, education) and compared the results with those of an Australian study that used the source HLQ. Similarities and differences in the observed relationships were discussed
4.3 Validity generalisation is established for a PROM that is translated across two or more cultures	Evidence of validity generalisation information to support valid score interpretation and use of translated PROMs in other cultures similar to those already studied. Validity generalisation relates the PROM constructs within a nomological net [18]. To the extent that the net is well established, coherent and in accord with theory, the PROM can be used cautiously in new contexts (settings, cultures) without full validation for each proposed interpretation and/or use in that new context	Systematic review of results of validity studies of translated PROM scales across the five categories of validity evidence in the *Standards* against argued criteria in related and unrelated settings and cultures. Specific targeted studies to investigate/establish validity generalisability. This has not yet been systematically conducted for the HLQ. As a start to this sort of research, the English HLQ [55, 73], as well as the Danish [60] and German [61] studies, could be considered mainstream populations, whereas the Slovak [62, 77, 79] translation is an example of a targeted study of validity generalisation in a specific population
**5. Validity and consequences of testing** [11]*—*the intended and unintended consequences of test use, and as traced to a source of invalidity such as construct underrepresentation or construct-irrelevant components		
5.1 PROM users (e.g. health professionals, researchers) interpret and use respondents’ scores from a translated PROM as intended by the developers of the source language PROM and for the intended benefit	Evidence that the intended benefit from testing with the translated PROM has been realised	In-depth interviews with users of a translated PROM to assess the outcomes that arose from testing with the translated PROM (i.e. predicted or actual actions taken from score interpretation and use) and if these align with the intended benefits, as stipulated by the developers of the source language PROM. For example, the OPtimising HEalth LIteracy and Access (Ophelia) process [73] supports healthcare services to interpret and apply data from the nine HLQ scales (supported by co-design research practices) to design health literacy interventions. Evaluation cycles integrated into the Ophelia process aim to keep the interventions directed towards client, health professional, service and community health literacy. A follow-up evaluation (yet to be conducted) could determine the degree to which the users’ HLQ score interpretations generated interventions that were implemented and that delivered the intended health literacy benefits
5.2 Claims for benefits of testing that are not based directly on the developers’ intended score interpretations and uses	Evidence to determine if there are potential testing benefits that go beyond the intended interpretation and use of the translated PROM scores. For example, the HLQ was not designed to measure the broad concept of patient experience. However, data from the HLQ could be used for this purpose because the constructs and items include information about this concept. Consequently, a hospital that sought to measure patients’ health literacy might also make claims about patients’ hospital experiences	A companion or follow-up study or a critical review by an external evaluator could identify and evaluate benefits that are directly based on intended score interpretation and use (as based on the source language PROM) and benefits that are based on grounds other than intended score interpretation and use. For example, a companion study could consist of co-administering the HLQ with specific patient experience questionnaires, auditing patient complaints records, and undertaking in-depth interviews with patients and hospital staff to determine the validity of HLQ score interpretation for measuring patient experiences
5.3 Awareness of and mitigation of unintended consequences of testing due to construct underrepresentation and/or construct irrelevance to prevent inappropriate decisions or claims about an individual or group, i.e. to take action/intervention when not warranted, or to take no action/intervention when an action is warranted, or to falsely claim an action/intervention is a success or a failure	Evidence of sound translation method to help minimise unintended consequences related to errors in score interpretation for a given use that are due to poor equivalence between the source language and translated PROM constructs Evidence of appropriate methods to calculate and interpret scale scores, including judgements about what are large, small or null differences between populations	Collection and analysis of translation process data that verify that a structured translation method is implemented such that congruence between source language and translated PROM constructs, and potential construct underrepresentation and/or construct irrelevance, is continually addressed. For example, an as yet unpublished study has been conducted to analyse field notes from translations of the HLQ into nine languages to determine aspects of the translation method that improve congruence of item intent between the source and translated items and thus constructs Cognitive interview testing of the translated items verifies the appropriateness of the translation for target respondents [60] Analysis of process data that a PROM output interpretation guide that prescribes data analysis methods to mitigate inappropriate data claims has been effectively used

## Discussion and conclusion

Validity theory and methodology, as based on the *Standards* and the work of Kane, provide a novel framework for determining the necessary validity testing for new PROMs or for PROMs in new contexts, and for making decisions about the validity of score inferences for use in these contexts. The first step in the process is to describe the proposed interpretive argument (including associated assumptions) for the PROM, and the second step is to collate (or generate) and evaluate the relevant evidence to establish a validity argument for the proposed interpretation and use of PROM scores. The *Standards* advocates that this iterative and cumulative process is the responsibility of the developer or user of a PROM for each new context in which the PROM is used (p. 13) [11]. Once the validity argument is as advanced as possible, the user is then required to make a judgement as to whether or not they can safely use the PROM for their intended purpose. The primary outcome of the process is a reasoned decision to use the PROM with confidence, use it with caveats, or to not use the PROM. This paper provides a theoretically sound framework for PROM developers and users for the iterative process of the validation of the inferences made from PROM data for a specific context. The framework guides PROM developers and users to assess the strengths of existing validity testing for a PROM, as well as to acknowledge gaps—articulated as caveats for interpretation and use—that can guide potential users of the PROM and future validity testing.

Validity theory, as outlined in the *Standards*, enables developers and users of PROMs to view validity testing in a new light: ‘This perspective has given rise to the situation wherein there is no singular source of evidence sufficient to support a validity claim’ (Chap. 1, p. 13) [10]. PROM validation is clearly much more than providing evidence for a type of validity; it is about systematically evaluating a range of sources of evidence to support a validity argument [10, 12, 19, 50]. It is also clearly insufficient to report only on selected statistical properties of a new PROM (e.g. reliability and factor structure) and claim the PROM is valid. Qualitative as well as quantitative research outputs are required to examine other aspects of a translated PROM, such as investigation of PROM translation methods [11]. Qualitative studies of translation methods enables insight into the target language words and phrases that are used by translators to convey the intended meaning of an item and that item’s relationship with the other items in its scale, with the scale’s response options, and with the construct it represents.

Evidence for the method of translation of a PROM to other languages is recommended by the *Standards* as part of a validity argument for a translated PROM [11]. Reviews have been done to describe common components of translation methods (e.g. use of forward and back translations, consensus meetings) [15, 83] and guidelines and recommendations are published [30, 76, 84] but qualitative studies that include examination of the core elements of a translation procedure are uncommon. It is critical that a PROM translation method can detect errors in the translation, can identify the introduction of linguistically correct but hard-to-understand wording in the target language, and can determine the acceptability of the underlying concepts to the local culture. The presence of a translation method, systematically assessed in the proposed framework for the process of validity testing, will assist PROM users to make better choices about the tools they use for research and practice.

Also required by the *Standards* as validity evidence is post-translation qualitative research into the response processes of people completing the translated PROM (i.e. the cognitive processes that occur when respondents formulate answers to the items) [5, 11]. Given the extensive and clear item intents for each HLQ item, cognitive interviews to investigate response processes would provide information, for example, about whether or not respondents in the target language formulate their responses to the items in line with the item intents and construct criteria [10, 56]. Castillo-Diaz and Padilla used cognitive interviews within the argument-based approach framework in order to obtain validity evidence about response processes [16]. Qualitative research can provide evidence, for example, about how a translation method or a new cultural context might alter respondent interpretation of PROM items and, consequently, their choice of answers to the items. This in turn influences the meaning derived from the scale scores by the user of the PROM. The decisions then made (i.e. the consequences of testing) might not be appropriate or beneficial to the recipients of the decision outcomes. Unfortunately, although qualitative investigations may accompany quantitative investigations of the development of new PROMs or use of a PROM in a new context, they are infrequently published as a form of PROM validity evidence [10].

Generating, assembling and interpreting validity evidence for a PROM requires considerable expertise and effort. This is a new process in the health sector and ways to accomplish it are yet to be explored. However, as outlined in this paper, it is important to undertake these tasks to ensure the integrity of the interpretations and corresponding decisions that are made from data derived from a PROM. The provision of easily accessible outcomes of argument-based validity assessment through publication would be welcomed by clinicians, policymakers, researchers, PROM developers and other users. The more evidence there is in the public domain for the use of the inferences made from a PROM’s data in different contexts, the more that users of the PROM can assess it for use in other contexts. This may reduce the burden on users needing to generate new evidence for each new interpretation and use. There may be cases where components of the five sources of evidence are necessary but not feasible. For example, the target population is narrowly defined and small in number (e.g. a minority language group as is used in our example) and large-scale quantitative testing is not possible. In such a case, the PROM may be able to be used but with caveats that data should be interpreted cautiously and decisions made with support from other sources (e.g. clinical expertise, feedback from community leaders). These sorts of concerns highlight the importance of establishing PROM validity generalisation (see Row 4.3 in Table 1) through building nomological networks of theory and evidence [18] that support interpretation for a broadening range of purposes. But the question that remains is who would be the custodian of such validity evidence? The way forward to promote and maintain improved validity practice in the PROM field may be through communities of practice or through repositories linked to specific organisations, institutions or researchers [85].

As far as we are aware, there are few publications in the health sector about the process of accumulating and evaluating evidence for a validity argument to support an intended interpretation and use of PROM data, an exception being Beauchamp and McEwan’s discussion about sources of evidence relating to response processes in self-report questionnaires in health psychology (Chap. 2, pp. 13–30) [5]. The application and adaptation of contemporary validity testing theory and an argument-based approach to validation for PROMs will support PROM developers and users to efficiently and comprehensively organise clear interpretive arguments and determine the required evidence to verify the use of one PROM over others, or to establish the strength of an interpretive argument for a particular PROM. The theoretical and methodological processes in this paper are offered as an advancement of the theory and practice of PROM validity testing in the health sector. These processes are intended as a way to improve PROM data and establish interpretations and decisions made from these data as compelling sources of information that contribute to our understanding of the well-being and health outcomes of our communities.

## Footnotes

1 There is some exchange in this paper between the terms ‘tool’ and ‘test’. The *Standards* refers to a ‘test’ and, when referencing the *Standards*, the authors will also refer to a ‘test’. The *Standards* is written primarily for educators and psychologists, professions in which testing students and clients, respectively, is undertaken. Patient-reported outcomes measures (PROMs) used in the field of health are not used in the same way as testing for educational grading or for psychological diagnosis. PROMs are primarily used to provide information about healthcare options or effectiveness of treatments.

2 We use ‘robust’ to describe the required psychometric properties of a PROM in the same way that it is used more generally in the test development and review literature to indicate (a) that in the development stage, a PROM achieves acceptable benchmarks across a range of relevant statistical tests (e.g. a composite reliability or Cronbach’s alpha of = > 0.8; a single-factor model for each scale in a multi-scale PROM giving satisfactory fit across a range of fit statistics, clear discrimination across these scales etc.) and (b) that these results are replicated (i.e. remain acceptably stable) across a range of different contexts and uses of the PROM.

## References

[1] Nelson EC, Eftimovska E, Lind C, Hager A, Wasson JH, Lindblad S (2015). Patient reported outcome measures in practice. BMJ.

[2] Williams K, Sansoni J, Morris D, Grootemaat P, Thompson C, ACSQHC (2016). Patient-reported outcome measures: Literature review.

[3] Ellwood PM (1988). Shattuck lecture—outcomes management: A technology of patient experience. New England Journal of Medicine.

[4] Marshall S, Haywood K, Fitzpatrick R (2006). Impact of patient-reported outcome measures on routine practice: A structured review. Journal of Evaluation in Clinical Practice.

[5] Zumbo BD, Hubley AM (2017). Understanding and investigating response processes in validation research (Vol.69). Social Indicators Research Series).

[6] Zumbo BD, Lissitz RW (2009). Validity as contextualised and pragmatic explanation, and its implications for validation practice. The concept of validity: Revisions, new directions, and applications.

[7] Thompson C, Sonsoni J, Morris D, Capell J, Williams K, ACSQHC (2016). Patient-reported outcomes measures: An environmental scan of the Australian healthcare sector.

[8] Lohr KN (2002). Assessing health status and quality-of-life instruments: Attributes and review criteria. Quality of Life Research.

[9] McClimans L (2010). A theoretical framework for patient-reported outcome measures. Theoretical Medicine and Bioethics.

[10] Zumbo BD, Chan EK (2014). Validity and validation in social, behavioral, and health sciences (Social Indicators Research Series.

[11] American Educational Research Association, American Psychological Association, & National Council on Measurement in Education (2014). Standards for educational and psychological testing.

[12] Kane M (2013). The argument-based approach to validation. School Psychology Review.

[13] Food US, Administration D, Center for Devices and Radiological Health (2009). Center for Drug Evaluation and Research, Center for Biologics Evaluation and Research. Guidance for industry: Patient-reported outcome measures: Use in medical product development to support labeling claimsFederal Register.

[14] Terwee CB, Mokkink LB, Knol DL, Ostelo RW, Bouter LM, de Vet HC (2012). Rating the methodological quality in systematic reviews of studies on measurement properties: A scoring system for the COSMIN checklist. Quality of Life Research.

[15] Reeve BB, Wyrwich KW, Wu AW, Velikova G, Terwee CB, Snyder CF (2013). ISOQOL recommends minimum standards for patient-reported outcome measures used in patient-centered outcomes and comparative effectiveness research. Quality of Life Research.

[16] Castillo-Díaz M, Padilla J-L (2013). How cognitive interviewing can provide validity evidence of the response processes to scale items. Social Indicators Research.

[17] Gadermann AM, Guhn M, Zumbo BD (2011). Investigating the substantive aspect of construct validity for the satisfaction with life scale adapted for children: A focus on cognitive processes. Social Indicators Research.

[18] Cronbach LJ, Meehl PE (1955). Construct validity in psychological tests. Psychological Bulletin.

[19] Messick S (1980). Test validity and the ethics of assessment. American Psychologist.

[20] Sireci SG (2007). On validity theory and test validation. Educational Researcher.

[21] Shepard LA (1997). The centrality of test use and consequences for test validity. Educational Measurement: Issues and Practice.

[22] Moss PA, Girard BJ, Haniford LC (2006). Validity in educational assessment. Review of Research in Education.

[23] Kane MT (1992). An argument-based approach to validity. Psychological Bulletin.

[24] Hubley AM, Zumbo BD (2011). Validity and the consequences of test interpretation and use. Social Indicators Research.

[25] Cronbach LJ, Thorndike RL, Angoff WH, Lindquist EF (1971). Test validation. Educational measurement.

[26] Anastasi A (1950). The concept of validity in the interpretation of test scores. Educational and Psychological Measurement.

[27] Nelson E, Hvitfeldt H, Reid R, Grossman D, Lindblad S, Mastanduno M (2012). ). Using patient-reported information to improve health outcomes and health care value: Case studies from Dartmouth, Karolinska and Group Health.

[28] Cook DA, Brydges R, Ginsburg S, Hatala R (2015). A contemporary approach to validity arguments: A practical guide to Kane’s framework. Medical Education.

[29] Moss PA (1998). The role of consequences in validity theory. Educational Measurement: Issues and Practice.

[30] Wild D, Grove A, Martin M, Eremenco S, McElroy S, Verjee-Lorenz A (2005). Principles of good practice for the translation and cultural adaptation process for patient-reported outcomes (PRO) measures: Report of the ISPOR Task Force for Translation and Cultural Adaptation. Value in Health.

[31] Buchbinder R, Batterham R, Elsworth G, Dionne CE, Irvin E, Osborne RH (2011). A validity-driven approach to the understanding of the personal and societal burden of low back pain: Development of a conceptual and measurement model. Arthritis Research & Therapy.

[32] Sireci SG (1998). The construct of content validity. Social Indicators Research.

[33] Shepard LA, Darling-Hammond L (1993). Evaluating test validity. Review of research in education.

[34] American Psychological Association, American Educational Research Association, & National Council on Measurement in Education (1954). Technical recommendations for psychological tests and diagnostic techniques.

[35] Camara WJ, Lane S (2006). A historical perspective and current views on the standards for educational and psychological testing. Educational Measurement: Issues and Practice.

[36] National Council on Measurement in Education (2017). Revision of the Standards for Educational and Psychological Testing. https://www.ncme.org/ncme/NCME/NCME/Resource_Center/Standards.aspx. Accessed 28 July 2017.

[37] American Psychological Association, American Educational Research Association, National Council on Measurement in Education, & American Educational Research Association Committee on Test Standards (1966). Standards for educational and psychological tests and manuals.

[38] Moss PA (2007). Reconstructing validity. Educational Researcher.

[39] American Psychological Association, American Educational Research Association, & National Council on Measurement in Education (1974). Standards for educational & psychological tests.

[40] Messick S (1995). Standards of validity and the validity of standards in performance asessment. Educational Measurement: Issues and Practice.

[41] Messick S (1995). Validity of psychological assessment: Validation of inferences from persons’ responses and performances as scientific inquiry into score meaning. American Psychologist.

[42] Messick S (1989). Meaning and values in test validation: The science and ethics of assessment. Educational Researcher.

[43] American Educational Research Association, American Psychological Association, & National Council on Measurement in Education (1985). Standards for educational and psychological testing.

[44] American Educational Research Association, American Psychological Association, Joint Committee on Standards for Educational and Psychological Testing (U.S.), & National Council on Measurement in Education (1999). Standards for educational and psychological testing.

[45] Kane MT (2013). Validating the interpretations and uses of test scores. Journal of Educational Measurement.

[46] Messick S (1990). Validity of test interpretation and use. ETS Research Report Series.

[47] Messick S (1992). The Interplay of Evidence and consequences in the validation of performance assessments.

[48] Litwin MS (1995). How to measure survey reliability and validity.

[49] McDowell I (2006). Measuring health: A guide to rating scales and questionnaires.

[50] Kane MT (1990). An argument-based approach to validation. ACT research report series.

[51] Kane M (2010). Validity and fairness. Language Testing.

[52] Caines J, Bridglall BL, Chatterji M (2014). Understanding validity and fairness issues in high-stakes individual testing situations. Quality Assurance in Education.

[53] Anthoine E, Moret L, Regnault A, Sébille V, Hardouin J-B (2014). Sample size used to validate a scale: A review of publications on newly-developed patient reported outcomes measures. Health and Quality of Life Outcomes.

[54] Nutbeam D (1998). Health promotion glossary. Health promotion international.

[55] Osborne RH, Batterham RW, Elsworth GR, Hawkins M, Buchbinder R (2013). The grounded psychometric development and initial validation of the Health Literacy Questionnaire (HLQ). BMC Public Health.

[56] Hawkins M, Gill SD, Batterham R, Elsworth GR, Osborne RH (2017). The Health Literacy Questionnaire (HLQ) at the patient-clinician interface: A qualitative study of what patients and clinicians mean by their HLQ scores. BMC Health Services Research.

[57] Beauchamp A, Buchbinder R, Dodson S, Batterham RW, Elsworth GR, McPhee C (2015). Distribution of health literacy strengths and weaknesses across socio-demographic groups: A cross-sectional survey using the Health Literacy Questionnaire (HLQ). BMC Public Health.

[58] Elsworth GR, Beauchamp A, Osborne RH (2016). Measuring health literacy in community agencies: A Bayesian study of the factor structure and measurement invariance of the health literacy questionnaire (HLQ). BMC Health Services Research.

[59] Morris RL, Soh S-E, Hill KD, Buchbinder R, Lowthian JA, Redfern J (2017). Measurement properties of the Health Literacy Questionnaire (HLQ) among older adults who present to the emergency department after a fall: A Rasch analysis. BMC Health Services Research.

[60] Maindal HT, Kayser L, Norgaard O, Bo A, Elsworth GR, Osborne RH (2016). Cultural adaptation and validation of the Health Literacy Questionnaire (HLQ): Robust nine-dimension Danish language confirmatory factor model. SpringerPlus.

[61] Nolte, S., Osborne, R. H., Dwinger, S., Elsworth, G. R., Conrad, M. L., Rose, M., et al. (2017). German translation, cultural adaptation, and validation of the Health Literacy Questionnaire (HLQ). *PLoS ONE*, 12(2), 10.1371/journal.pone.0172340. 10.1371/journal.pone.0172340 PMC5325258 28234987

[62] Kolarčik P, Cepova E, Geckova AM, Elsworth GR, Batterham RW, Osborne RH (2017). Structural properties and psychometric improvements of the health literacy questionnaire in a Slovak population. International Journal of Public Health.

[63] Busija L, Buchbinder R, Osborne R (2016). Development and preliminary evaluation of the OsteoArthritis Questionnaire (OA-Quest): A psychometric study. Osteoarthritis and Cartilage.

[64] Batterham RW, Buchbinder R, Beauchamp A, Dodson S, Elsworth GR, Osborne RH (2014). The OPtimising HEalth LIterAcy (Ophelia) process: Study protocol for using health literacy profiling and community engagement to create and implement health reform. BMC Public Health.

[65] Friis K, Lasgaard M, Osborne RH, Maindal HT (2016). Gaps in understanding health and engagement with healthcare providers across common long-term conditions: A population survey of health literacy in 29 473 Danish citizens. British Medical Journal Open.

[66] Lim S, Beauchamp A, Dodson S, McPhee C, Fulton A, Wildey C (2017). Health literacy and fruit and vegetable intake. Public Health Nutrition.

[67] Griva K, Mooppil N, Khoo E, Lee VYW, Kang AWC, Newman SP (2015). Improving outcomes in patients with coexisting multimorbid conditions—the development and evaluation of the combined diabetes and renal control trial (C-DIRECT): Study protocol. British Medical Journal Open.

[68] Morris, R., Brand, C., Hill, K. D., Ayton, D., Redfern, J., Nyman, S., et al. (2014). RESPOND: A patient-centred programme to prevent secondary falls in older people presenting to the emergency department with a fall—protocol for a mixed methods programme evaluation. *Injury Prevention*, injuryprev-2014-041453. 10.1136/injuryprev-2014-041453 25392367

[69] Redfern J, Usherwood T, Harris M, Rodgers A, Hayman N, Panaretto K (2014). A randomised controlled trial of a consumer-focused e-health strategy for cardiovascular risk management in primary care: The Consumer Navigation of Electronic Cardiovascular Tools (CONNECT) study protocol. British Medical Journal Open.

[70] Banbury A, Parkinson L, Nancarrow S, Dart J, Gray L, Buckley J (2014). Multi-site videoconferencing for home-based education of older people with chronic conditions: The Telehealth Literacy Project. Journal of Telemedicine and Telecare.

[71] Faruqi N, Stocks N, Spooner C, el Haddad N, Harris MF (2015). Research protocol: Management of obesity in patients with low health literacy in primary health care. BMC Obesity.

[72] Livingston PM, Osborne RH, Botti M, Mihalopoulos C, McGuigan S, Heckel L (2014). Efficacy and cost-effectiveness of an outcall program to reduce carer burden and depression among carers of cancer patients [PROTECT]: Rationale and design of a randomized controlled trial. BMC Health Services Research.

[73] Beauchamp A, Batterham RW, Dodson S, Astbury B, Elsworth GR, McPhee C (2017). Systematic development and implementation of interventions to Optimise Health Literacy and Access (Ophelia). BMC Public Health.

[74] Osborne RH, Elsworth GR, Whitfield K (2007). The Health Education Impact Questionnaire (heiQ): An outcomes and evaluation measure for patient education and self-management interventions for people with chronic conditions. Patient Education and Counseling.

[75] Osborne RH, Norquist JM, Elsworth GR, Busija L, Mehta V, Herring T (2011). Development and validation of the influenza intensity and impact questionnaire (FluiiQ™). Value in Health.

[76] Rabin R, Gudex C, Selai C, Herdman M (2014). From translation to version management: A history and review of methods for the cultural adaptation of the EuroQol five-dimensional questionnaire. Value in Health.

[77] Kolarčik P, Čepová E, Gecková AM, Tavel P, Osborne R (2015). Validation of Slovak version of Health Literacy Questionnaire. The European Journal of Public Health.

[78] Vamos S, Yeung P, Bruckermann T, Moselen EF, Dixon R, Osborne RH (2016). Exploring health literacy profiles of Texas university students. Health Behavior and Policy Review.

[79] Kolarčik P, Belak A, Osborne RH (2015). The Ophelia (OPtimise HEalth LIteracy and Access) Process. Using health literacy alongside grounded and participatory approaches to develop interventions in partnership with marginalised populations. European Health Psychologist.

[80] Fornell C, Larcker DF (1981). Evaluating structural equation models with unobservable variables and measurement error. Journal of Marketing Research.

[81] Farrell AM (2010). Insufficient discriminant validity: A comment on Bove, Pervan, Beatty. and Shiu (2009). Journal of Business Research.

[82] Campbell DT, Fiske DW (1959). Convergent and discriminant validation by the multitrait-multimethod matrix. Psychological Bulletin.

[83] Epstein J, Santo RM, Guillemin F (2015). A review of guidelines for cross-cultural adaptation of questionnaires could not bring out a consensus. Journal of Clinical Epidemiology.

[84] Kuliś D, Bottomley A, Velikova G, Greimel E, Koller M (2016). EORTC quality of life group translation procedure.

[85] Enright M, Tyson E (2011). Validity evidence supporting the interpretation and use of TOEFL iBT scores.

